# Resilience and personality as predictors of the biological stress load during the first wave of the Covid-19 pandemic in Germany

**DOI:** 10.1038/s41398-021-01569-3

**Published:** 2021-08-28

**Authors:** Veronika Engert, Jost U. Blasberg, Sophie Köhne, Bernhard Strauss, Jenny Rosendahl

**Affiliations:** 1 Institute of Psychosocial Medicine, Psychotherapy and Psychooncology, Jena University Hospital, Friedrich Schiller University, Jena, Germany; 2 Research Group Social Stress and Family Health, Max Planck Institute for Human Cognitive and Brain Sciences, Leipzig, Germany

**Keywords:** Predictive markers, Human behaviour

## Abstract

Since the Covid-19 outbreak, pandemic-specific stressors have potentiated the—already severe—stress load across the world. However, stress is more than an adverse state, and chronic exposure is causally involved in the development of mental and physical disease. We ask the question whether resilience and the Big Five personality traits predict the biological stress response to the first lockdown in Germany. In a prospective, longitudinal, observational study, *N* = 80 adult volunteers completed an internet-based survey prior to the first Covid-19-related fatality in Germany (T0), during the first lockdown period (T1), and during the subsequent period of contact restrictions (T2). Hair strands for the assessment of systemic cortisol and cortisone levels were collected at T2. Higher neuroticism predicted higher hair cortisol, cortisone and subjective stress levels. Higher extraversion predicted higher hair cortisone levels. Resilience showed no effects on subjective or physiological stress markers. Our study provides longitudinal evidence that neuroticism and extraversion have predictive utility for the accumulation of biological stress over the course of the pandemic. While in pre-pandemic times individuals high in neuroticism are typically at risk for worse health outcomes, extraverted individuals tend to be protected. We conclude that, in the pandemic context, we cannot simply generalize from pre-pandemic knowledge. Neurotic individuals may currently suffer due to their general emotional lability. Extraverted individuals may primarily be socially stressed. Individualized stress management programs need to be developed, and offered in a lockdown-friendly format, to minimize the stress burden caused by Covid-19 or future pandemics and to protect the most severely affected individuals from the development of stress-associated disease.

## Introduction

More than a year has passed since the World Health Organization declared the new coronavirus (severe acute respiratory syndrome coronavirus 2), first reported in Wuhan in the Hubei Province of China, a pandemic (on 03/11/2020) [1]. Since then, our lives have changed dramatically, dominated by novelty, unpredictability, and a severe loss of control. Due to these very experiences, the current global health crisis is also a global stress crisis. Next to the fear of infection, and resulting from efforts to limit the spread of the virus, people are fearing for their jobs, bearing financial losses, and suffering from the increased burden of childcare, often while completing their normal work hours. Loneliness and feeling constrained in one’s home environment are posing additional emotional strain. There is little doubt that any one of these factors would increase the experienced stress load. Data gathered since the outbreak of the pandemic confirm this assumption, showing elevated levels of subjective stress and stress-associated emotional deterioration [2–6]. Such data provide valuable insights into the coronavirus disease 2019 (Covid-19)-related stress burden. However, in stress research, self-reports are not reliably linked to bodily processes [7–9], which may be due to known biases in self-report methods [10, 11]. To identify the individuals most at risk for developing long-term stress-associated health impairments, we need to factor in stress physiology.

Stress refers to a state in which adverse stimuli threaten an organism’s homeostasis [12, 13]. The organism subsequently generates a compensatory response of sympathetic–adrenal–medullary system and hypothalamic–pituitary–adrenal (HPA) axis activation. As a result, catecholamine and cortisol are released and trigger downstream effects on metabolic, cardiovascular, immune, and gastrointestinal functions, among others [12, 13]. In the acutely threatening event, this active process termed allostasis is a highly adaptive cascade of physiological events providing the organism with the necessary motivation and energy to survive [14, 15]. However, if activated over an extended period of time, a wear and tear on the body, termed allostatic load may accumulate [14, 15], leading to the development of prevalent medical conditions, such as mood disorders, cardiovascular, metabolic, gastrointestinal, and autoimmune diseases [12, 16]. To prevent such adverse health effects due to pandemic-specific chronic stress, healthcare systems need to prepare for future pandemics or imminent waves of the current one. Identifying individual risk and protective factors for the accumulation of allostatic load over the course of this crisis will be requisite to finding individualized and targeted interventions of stress reduction.

Resilience is discussed as a pivotal capacity to cope with stress and adversity during the Covid-19 pandemic [17]. In a definition derived from an extensive review of the literature, covering resilience both as a trait and dynamic process, it is broadly described as the capacity to adapt to significant experiences of stress or trauma [18]. Next to resources within the individual, life and environmental features contribute to this capacity to “bounce back” in the face of adversity [18]. Irrespective of whether conceptualized as a process [19] or trait [20], resilience is linked to good mental health [21, 22]. Also, it shows reliable associations with personality traits, such that resilient individuals exhibit lower levels of neuroticism, and higher levels of extraversion, openness, agreeableness, and conscientiousness [23].

Launched in the early phase of the pandemic, a cross-sectional online survey conducted in 24 languages in almost 16,000 adults confirmed the utility of a resilience-focused approach to understanding the psychological consequences of the Covid-19 pandemic [6]. Data from this study identified positive appraisal style and good recovery from stress as the strongest factors in resilience, defined by the authors as the maintenance of health despite adversity [19]. Operationalized as a trait, resilience was shown to mediate the effects of personality traits, that is, neuroticism, openness, agreeableness, and conscientiousness, on stress and subjective well-being experienced at the beginning of the pandemic. Neuroticism was identified as the strongest predictor of maladaptive psychological functioning, both directly and via diminished resilience [24].

The impact of personality traits on psychological adaptation to the pandemic was also investigated independent of resilience and yielded consistent results only for agreeableness and neuroticism. While agreeableness was linked to better psychological adjustment and lower stress [25, 26], neuroticism was linked to an overall worse outcome [5, 25–31] (more detailed results on the study outcomes are summarized in Table 1). Extraversion, openness, and conscientiousness held an ambiguous position, identified as both risk and protective factors [5, 25, 26, 31–33] (Table 1).

**Table 1:** Current studies on associations of the Big Five personality traits with psychological adjustment and stress since outbreak of the Covid-19 pandemic.

Authors	Traits	Risk
Aschwanden et al. [27]	Neuroticism	More concerns
Fernández et al. [28]	Neuroticism	Psychological distress
Gubler et al. [29]	Neuroticism	Higher loneliness and lower well-being
Kroencke et al. [30]	Neuroticism	Higher negative affect and crisis preoccupation
Liu et al. [32]	Extraversion	Higher stress
Nikčević et al. [25]	Neuroticism	Higher generalized anxiety and depressive symptoms
Qian and Yahara [26]	Neuroticism	Higher stress, anxiety, depression, more family-specific concerns
	Extraversion	More family-specific concerns
	Openness	Higher stress
Robillard et al. [5]	Neuroticism	Higher stress
	Extraversion	
	Conscientiousness	
Zacher and Rudolph [31]	Neuroticism	Higher stress
	Extraversion	

		Protection
Morales-Vives et al. [33]	Extraversion	Better adaptation
Nikčević et al. [25]	Extraversion	Lower generalized anxiety and depressive symptoms
	Openness	
	Agreeableness	
	Conscientiousness	
Qian and Yahara [26]	Openness	Less family-specific concerns
	Agreeableness	Lower stress and anxiety
	Conscientiousness	Lower depression

Note: Risk: worse psychological adjustment, higher stress; protection: better psychological adjustment, lower stress.

**Fig. 1:**
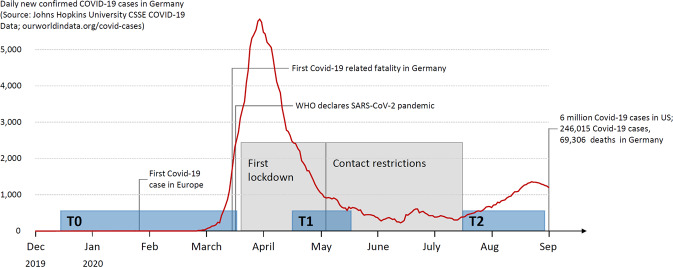
Testing timeline relative to important milestones in the pandemic timeline in Germany. Data collection took place before and during the very early stages of the Covid-19 pandemic, prior to the first Covid-19-related fatality in Germany (T0: 12/14/2019–03/10/2020), during the first lockdown period (T1: 04/11/2020–05/08/2020), and during the subsequent period of contact restrictions (T2: 07/10/2020–08/27/2020).

Building on these self-report studies, we examined in a sample of *N* = 80 healthy adults whether resilience and personality predicted participants’ hormonal stress responses to the first lockdown (03/22/2020–05/03/2020) and subsequent period of contact restrictions in Germany (until mid-July 2020). Accordingly, levels of cortisol and cortisone in hair were examined as biomarkers of long-term stress. Determined by an average hair growth rate of one cm per month, 3 cm hair segments capture the systemic hormone exposure over the past 3 months and are linked to the subjective experience of psychosocial stress over the same timeframe [34]. With data collection stretching from mid-July to mid-August 2020, our measurement reflects cortisol/cortisone exposure starting between mid-April to mid-May (see Fig. 1 for an overview of the testing timeline). Spanning the month prior to the assessment, retrospective self-reports of stress were captured using the Perceived Stress Scale (PSS) [35]. Personality as a stable trait was assessed once (between mid-December 2019 and mid-March 2020), before the first Covid-19-related fatality in Germany (on 03/08/2020), using the NEO Five Factor Inventory (NEO-FFI) [36]. Resilience, conceptualized both as trait and dynamic state, was assessed at this early measurement time point using the Resilience Scale (RS) [20] and the Brief Resilience Scale (BRS) [37]. State resilience was collected for a second time 4 weeks into the lockdown (mid-April 2020). This repeated measurement allowed gauging adaptation to the first period of severe lockdown restrictions.

We predicted to find lower hair cortisol, cortisone, and subjective stress levels, indicating a relatively reduced stress burden during the lockdown period, in participants with higher trait resilience and following a rise in resilience from before to within lockdown. Relatively higher scores in neuroticism were contrarily expected to predict an increased stress load. Given the sparsity and heterogeneity of Covid-19-specific results for the remaining Big Five personality traits, we suggest that typical associations with stress and psychological well-being may have shifted since the onset of the pandemic. Therefore, instead of specifying a priori hypotheses, we took an exploratory perspective to determine which of the current self-report results is corroborated by biological data.

## Methods

### Study design

This is a prospective, longitudinal, observational study with three measurement time points assessed before and during the Covid-19 pandemic in 2020.

### Setting and participants

Adult volunteers in Germany were invited to complete an internet-based survey at two measurement time points (T0, T1). In light of the unfolding pandemic situation, we asked participants to additionally rate subjective stress and provide hair strands for the assessment of systemic cortisol and cortisone levels at a third measurement time point (T2). Apart from adult age, no inclusion criteria were defined; therefore, participants did not disclose information on (psycho-)pathology, medication intake, or drug abuse. The present research was designed in accordance with the Declaration of Helsinki and approved by the ethics committee of the Friedrich Schiller University Jena, Germany (#2019-1609-Bef, 12/12/2019 and #2019-1609_1-Bef, 02/07/2020).

Data collection took place before and during the very early stages of the Covid-19 pandemic, prior to the first Covid-19-related fatality in Germany (T0: 12/14/2019–03/10/2020), during the first lockdown period (T1: 04/11/2020–05/08/2020), and during the subsequent period of contact restrictions (T2: 07/10/2020–08/27/2020) relative to important milestones of the pandemic progression in Germany (see Fig. 1 for an overview of the testing timeline). Information about the study was distributed via advertisement on several online sites, including the official Facebook page for press releases of Jena University Hospital (>11,000 followers), various student and regional Facebook interest groups, the website of a leading magazine for psychology and related disciplines (psychologie-heute.de), and via snowball principle. After termination of the first survey, we asked participants to provide their email addresses for invitation to subsequent data assessments. To allow providing participants with all materials needed for hair sampling, they were asked to disclose their postal addresses. Participants provided written informed consent and were financially compensated (EUR 10) for their participation after the last measurement time point.

### Measures

#### Personality characteristics

At the baseline, pre-pandemic measurement time point (T0), we assessed the Big Five personality traits extraversion, neuroticism, agreeableness, conscientiousness, and openness to experience, using a German brief version [38] of the NEO-FFI-30 [36]. More detailed information on this and all subsequent questionnaires is given in the Supplementary Methods.

#### Resilience

We applied two measures of resilience at the baseline (T0) and second measurement time points (T1). The RS-25 [20] in its German version [39] was used to assess resilience as a positive personality characteristic that enhances individual adaption. The BRS [37] in its German version [40] assesses resilience as a dynamic state, targeting the ability to “bounce back” or recover from ongoing stressors.

#### Subjective stress

At the third measurement time point (T2), we used the German version of the PSS [35, 41] to assess the degree to which participants experienced their life as unpredictable, uncontrollable, and overloaded in the past month.

#### Hair cortisol and cortisone concentrations

At the third measurement time point (T2), levels of cortisol and its inactive metabolite and precursor molecule, cortisone, in hair were assessed. Both hair cortisol and cortisone concentrations are indicative of systemic glucocorticoid exposure and markers of chronic stress [34]. Hair cortisone levels have been suggested to yield a complementary, potentially more stable, glucocorticoid signal alongside cortisol itself [42]. While the precise mechanism behind hair cortisol and cortisone accumulation is incompletely understood, it is assumed that, during hair growth, free cortisol and cortisone molecules are continuously incorporated into hair follicles, proportional to their overall concentration in the physiological system. Hormone concentrations in a 1 cm hair segment are thus assumed to indicate the cumulative systemic cortisol/cortisone exposure over an approximately 1-month period [34]. For more detailed information on the hair sampling and analysis procedures, see Supplementary Methods.

### Bias

In survey studies, sampling biases (e.g., self-selection bias) may impact on the external validity of results. To examine the potential influence of self-selection, we compared our sample to existing normative data (NEO-FFI-30 [38]; RS-25 [39]; BRS [40]). We further attempted to prevent attrition bias by providing reminders for the completion of all survey items in case of incomplete data.

### Study size

An a priori power analysis was performed using G*Power 3.1 [43]. For a power of 0.9 at an alpha level of 0.05, a medium effect size of *f*^2^ = 0.15, and a maximum of 9 predictors (all covariates and the state resilience baseline × change interaction), a minimum sample size of *N* = 73 was calculated.

### Quantitative variables

Hair cortisol and cortisone data were log-transformed and winsorized to 3 SD to account for skewness. The PSS sum score was checked for normal distribution using Shapiro–Wilk’s test (*W* = 0.97, *p* value = 0.10), which suggested no significant diversion from the null hypothesis. For the dynamic resilience (BRS) measure, a change score between the first and second measurement time points (T1–T0) was calculated. Trait resilience, state resilience, NEO-FFI sum scores, and age were *z*-standardized to handle multi-collinearity.

### Statistical methods

All analyses were performed using R 4.0.3 [44]. Hair cortisol, cortisone, and PSS scores were entered into multiple linear regression models as outcome variables. Models were built upon age as a control variable and an interaction of baseline state resilience with the state resilience change score, thus controlling for the effect of the baseline measurement on change. Subsequently, the NEO-FFI scales were added iteratively, and each resulting model was compared to the previous, simpler model by means of model fit comparison using analyses of variance. Thus, only variables and interactions between state resilience and NEO-FFI scales that significantly improved model fit were retained, while keeping model complexity at the minimum. This modeling approach closely followed our theoretical assumptions, while retaining best possible model fit. For all relevant effects, standardized *β-*coefficients and confidence intervals (CIs) are reported.

In addition, the described multiple linear regression analyses were rerun with trait resilience as the relevant predictor next to personality traits. Since we did not expect trait resilience to fluctuate over time, it was only assessed at the initial measurement time point (T0).

Out of 82 participants who provided a hair sample, 2 cases with missing data in NEO-FFI scores were excluded. Because hair cortisol has been found to be unaffected [45–47], and our sample size only allowed for a limited amount of predictors, smoking and use of hormonal contraceptives in women were not entered as control variables. Furthermore, because the sample consisted mostly of women (72 out of 80 participants; 90%), and participants were highly educated overall (62 out of 80 participants finished high school, technical high school, college, or university; 77.5%), participant sex and level of education could not be added as control variables due to uneven distributions in the sample (see Table 2). To verify the robustness of our results despite the uneven number of male/female participants, we performed sensitivity analyses including only women.

**Table 2:** Characteristics of the final sample and comparison to normative values (*N* = 80).

			*n*	%
Gender (female)			72	90
Education				
Student			1	1.25
Dropped out of school			0	0
Completed basic secondary school (Hauptschule)			1	1.25
Completed secondary school (Realschule)			4	5
Completed technical high school (Fachhochschule)			6	7.5
Completed high school (Gymnasium)			22	27.5
Completed apprenticeship (Lehre)			9	11.25
Completed college/university			34	42.5
Other			3	3.75
Employment				
Student (high school)			1	1.25
Apprentice			1	1.25
College/university student			20	25
Employee			49	61.25
Public service official			2	2.5
Self-employed			1	1.25
Unemployed			0	0
Other			6	7.5

	*M*	SD	Difference to normative data (Cohen’s *d* with 95% CI)	
Age	35.65	11.49		
Personality traits (NEO-FFI)				
Extraversion	1.72	0.87	−0.33, CI −0.80 to 0.14	
Neuroticism	2.04	0.65	0.12, CI −0.36 to 0.59	
Agreeableness	3.10	0.53	0.45, CI −0.021 to 0.93	
Conscientiousness	3.05	0.57	0.14, CI −0.33 to 0.61	
Openness to experience	2.71	0.74	0.89, CI 0.40 to 1.38	
Resilience (BRS)	3.12	0.93	−0.39, CI −0.80 to 0.10	
Trait resilience (RS)	134.06	20.38	0.08, CI −0.36 to 0.53	

Because our sample consisted of mostly women (*N* = 72, 90%), for all measures except BRS, comparisons are based on previously reported normative data of women. Negative values of Cohen’s *d* indicate lower scores than the normative sample, positive values represent high scores.

*M* mean, *SD* standard deviation.

## Results

### Descriptive data

**Fig. 2:**
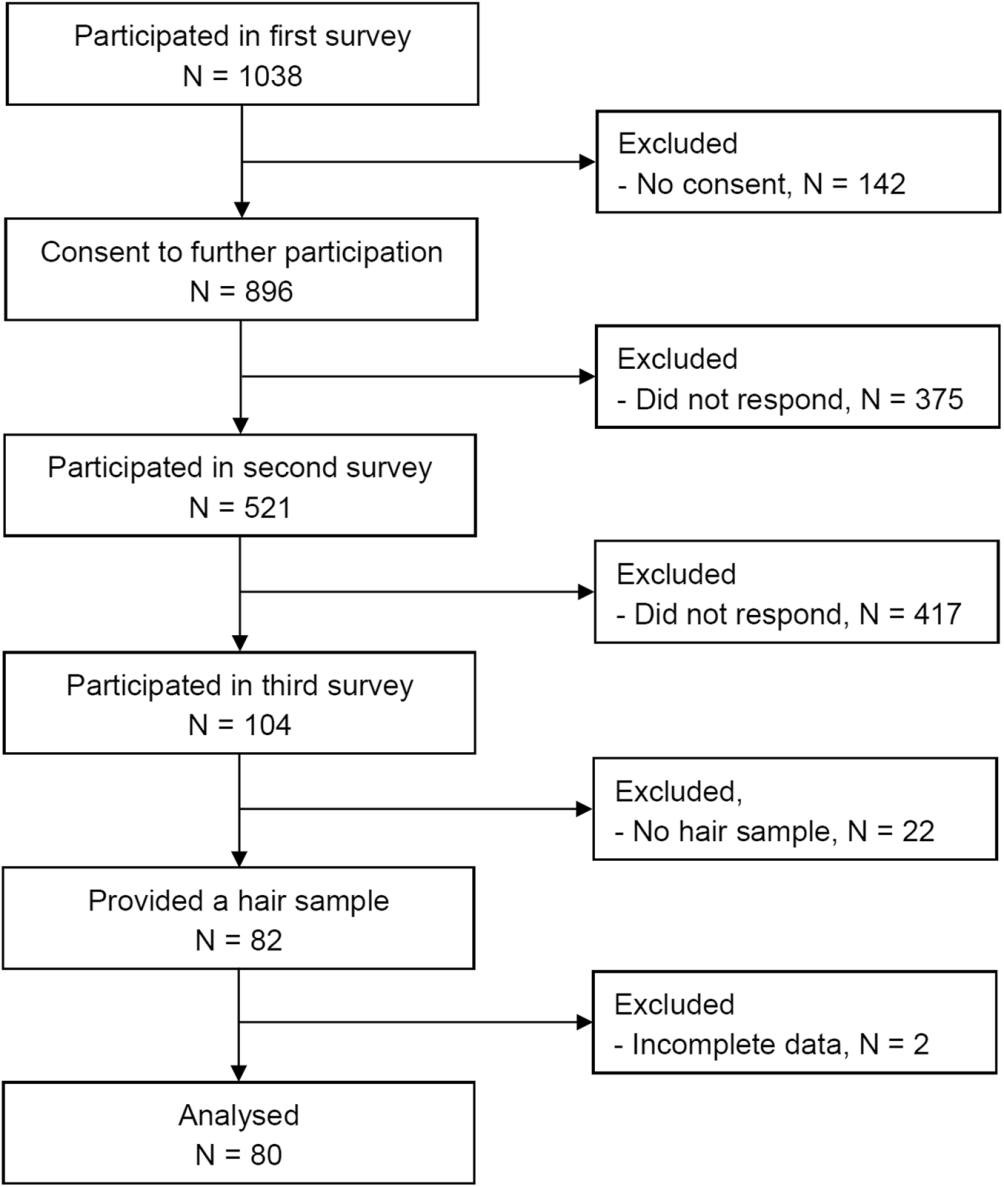
Flow chart of study inclusion. At T0, 1038 volunteers participated, of whom 521 were reassessed at T1 (50.2%), and 104 were assessed at T2 (10% of the initial sample size). *N* = 82 participants provided a hair sample. Because of missing questionnaire data, *N* = 80 participants were included in the final analysis; 72 (90%) were female; mean age was 35.65 years.

The analyzed sample is comprised of *N* = 80 participants (72 women; age *M* = 35.65, SD = 11.49, range = 17–66; see Fig. 2 for a diagram of participant flow from T0 to T2). Mean values in resilience and personality traits did not differ significantly from normative data, except for openness for experience, for which our participants scored significantly higher (Table 2). Means, standard deviations, and correlations of outcome variables are summarized in Supplementary Table S1. Levels of hair cortisol and hair cortisone showed no significant correlations with the PSS score. Hair cortisol and cortisone were highly correlated (*r* = 0.89, 95% CI 0.84 to 0.93). The *z*-standardized mean change in state resilience from T0 to T1 (*M* = −0.196, SD = 1.04) was insignificant when compared to a null hypothesis (*t*(79) = −0.373, 95% CI −0.25 to 0.17, *p* = 0.71).

### Main results

**Fig. 3:**
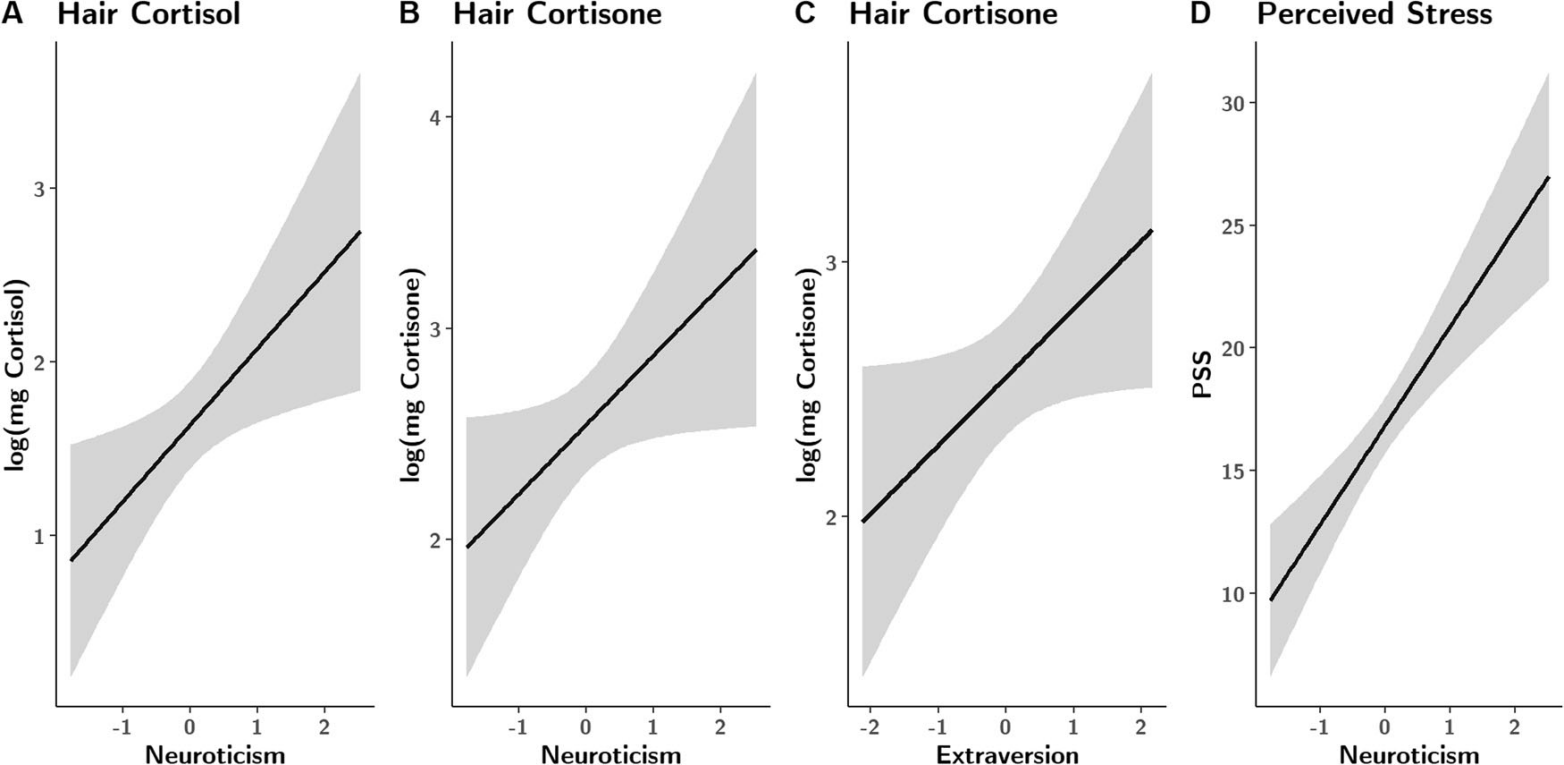
Fixed effects of multiple regression covariates. Estimated effects (and SD) of neuroticism and extraversion on hair cortisol (**A**), hair cortisone (**B**, **C**) and PSS scores (**D**). Neuroticism revealed positive associations across all stress markers, suggesting increasing physiological and subjective strain with increasing neuroticism. Extraversion had a significant effect on hair cortisone, also revealing a positive association.

A significant effect of neuroticism on hair cortisol was found (*β* = 0.37, 95% CI 0.08–0.66, *p* = 0.014), such that higher values of neuroticism predicted increasing levels of hair cortisol (Fig. 3A). Age also showed a significant effect (*β* = 0.28, 95% CI 0.07–0.50, *p* = 0.011), indicating higher hair cortisol with older age.

In terms of hair cortisone, neuroticism (*β* = 0.30, 95% CI 0.01–0.60, *p* = 0.045) and extraversion (*β* *=* 0.25, 95% CI 0.00–0.50, *p* = 0.049) revealed significant effects. Higher values in both personality traits predicted increased hair cortisone concentrations (Fig. 3B, C). Age again showed a significant effect (*β* *=* 0.26, 95% CI 0.04–0.49, *p* = 0.020). Neuroticism was also a significant predictor for self-reported stress (*β* *=* 0.61, 95% CI 0.36–0.85, *p* < 0.001), with higher values linked to a higher subjective stress load (Fig. 3D). All model estimates and indices can be found in the Supplementary Table S2.

Neither baseline values nor change in state resilience had an effect on any of the assessed stress markers. Detailed results from the additionally calculated trait resilience models are presented in the Supplementary Results and Table S3. Overall, trait resilience models showed weaker model fit, and again, resilience had no effect on the assessed stress markers. Otherwise, results mostly reflected the state resilience multiple regressions, with effects of extraversion on cortisone (*β* = 0.27, 95% CI 0.01–0.54, *p* = 0.042) and of neuroticism on perceived stress (*β* = 0.54, 95% CI 0.29–0.78, *p* < 0.001).

### Other analyses

Due to the high percentage of women in our sample, sensitivity analyses excluding all male participants were performed and largely confirmed the findings of our main analyses. Model estimates and results are presented in the Supplementary Tables S4 and S5.

## Discussion

Studies have shown that many people feel anxious and stressed in the ongoing Covid-19 pandemic [2, 4, 6]. The experience of stress, particularly over an extended time period, and if accompanied by the excessive release of the main stress hormone cortisol, is an important contributor to the development of disease, including mental disorders [12]. Accordingly, elevated levels of stress during the pandemic (due to fear of infection, and the negative consequences of the imposed confinement measures), are expected to contribute to widespread emotional strain and an increased risk for psychiatric illness [48]. In searching for potential risk and protective factors for an increased bodily stress load, we show that higher scores on the personality traits of neuroticism and extraversion predisposed individuals to a higher accumulation of cortisone in hair during the pandemic. Increased neuroticism also predicted increased hair cortisol and subjective stress levels. The other personality traits and resilience, irrespective of its conceptualization as a dynamic state or trait, had no influence on any of the stress markers.

The main contribution of the current study is its focus on an objective, biological marker of long-term stress. Our results on the biological stress load confirm and corroborate prior studies focusing solely on self-reports of pandemic stress. These studies consistently identified neuroticism as a predictor of maladaptive psychological functioning and stress [5, 25–31]. Likewise, outside the pandemic context, neuroticism is known as a personality trait of profound public health significance [49]. Understood as the tendency to respond with negative emotions to threat, frustration, or loss [36, 49], it shows stable associations with a wide variety of both mental and physical health problems [49], as well as increased stress sensitivity [50, 51].

Other than neuroticism, extraversion does not usually stand out as a risk factor. To the contrary, this tendency to be sociable, assertive, active, and positive [36, 52] is more often linked to positive health outcomes and attenuated stress experience [53]. Yet, in the context of the Covid-19 pandemic, extraversion was repeatedly associated with higher self-perceived stress [5, 31, 32]. Our data, showing that extraverts also exhibit elevated hair cortisone concentrations during and shortly after the first lockdown, extend this finding to the level of a biological stress marker. The pandemic situation seems to create challenges that are distinct from those of life as we know it. Particularly the social distancing measures may contribute to an increased stress load in individuals seeking a social and active lifestyle. Likely reflecting their difficulty to reduce social proximity, extraverted individuals also reported lower compliance with the social distancing measures in a Brazilian survey [54]. We suggest that neurotic and extraverted individuals suffer from different stress qualities during the pandemic. While neuroticism may predispose to emotional lability in general, high levels of extraversion may lead to social deprivation; the very strategies that are typically employed to buffer stress cannot be carried out.

Multiple factors could explain why we found no influence of trait or state resilience on the assessed stress markers. For one thing, given the minimal change in state resilience from T0 to T1, the calculated delta score was likely not a sensitive measure for dynamic resilience fluctuations. The lack of significant effects may likewise be an issue of statistical power or else of construct operationalization [i.e., especially the study by Veer et al. [6] had considerably more statistical power and operationalized resilience as an *outcome*]. However, there is also prior evidence from research in children and adolescents showing that resilience scales do not explain additional variance in emotional disturbance and adaptation, once the effect of the Big Five personality dimensions have been accounted for [55, 56]. It is thus possible, that above and beyond the variance explained by neuroticism and extraversion, resilience made no additional contribution to the pandemic stress load.

We found no association between hair glucocorticoid levels and subjective stress measured with the PSS. While an association would be expected given that both variables capture aspects of the construct stress, this lack of psychoendocrine covariance is a recurring phenomenon in stress research [7–9]. It may be particularly pronounced due to biases in retrospective self-reports [10, 11] and the fact that a considerable proportion of variance in hair glucocorticoid levels is attributable to stress-independent variables, such as a person’s general propensity to release glucocorticoids [57]. Also, an improvement in covariance by means of time-sensitive analysis techniques [9] is obviously precluded due to the integrative nature of hair glucocorticoid levels. In general, it may be a promising remedy to predict hair glucocorticoids in healthy adults through a combination of more objective self-report data, such as counts of daily hassles, and advanced statistical modeling of dynamic time courses in self-reported stress [57].

Several limitations of the current study need to be addressed. First, while we have a longitudinal study design, stress markers were only assessed once, at the final measurement time point. Therefore, pandemic-induced changes in stress experience could not be captured. With the ongoing progression of the pandemic, future studies will be in the position to repeatedly assess cortisol data and investigate pandemic-induced change in HPA axis activation. Second, small sample size may have led to type II errors (i.e., incorrectly accepting the null hypothesis). Third, as is common in survey studies, self-selection bias may have influenced the external validity of our results. The fact that 90% of our sample were women, on the other hand, may have limited generalizability. A high percentage of female responders was already apparent at T0 (81%). Extremely short hair or baldness in men caused additional dropout at T2. Importantly, however, personality and resilience of included participants were comparable to existing normative data, except for openness to experience, for which our sample showed significantly higher scores than the normative sample. This deviation from normality could be ascribed to a decreased probability of dropout in longitudinal data assessments for individuals with relatively increased openness [58].

The Covid-19 pandemic is the defining global health crisis of our time. We here show that personality traits have predictive utility for biological stress over the course of the pandemic. Specifically, neuroticism and extraversion are risk factors for the accumulation of allostatic load, measured in terms of hair cortisol and cortisone concentrations. While it is established that individuals high in neuroticism are at risk for worse health outcomes, extraverted individuals are typically protected. This illustrates that, in the pandemic context, we cannot a priori generalize our knowledge from pre-pandemic times and that the types of stress felt by neurotic and extraverted individuals may differ substantially. To minimize the stress burden caused by Covid-19 and protect the most severely affected individuals from the development of stress-associated disease, individualized stress management programs need to be developed and offered in a lockdown-friendly format. Individuals high in extraversion, for example, may benefit particularly from online group counseling and therapy sessions (see a recent meta-analysis and commentary on the efficiency of psychosocial (group) interventions in improving stress-associated immune system function) [59, 60]. Considering that the public health significance of any given trait depends on its ability to predict future adverse outcomes [49], the longitudinal evidence we provide is particularly relevant to identify neuroticism and extraversion as risk factors for adverse, stress-related consequences of the Covid-19 pandemic.

## Supplementary information

Supplemental Material
