# Supplementary material for: Resilience and personality as predictors of the biological stress load during the first wave of the Covid-19 pandemic in Germany
Source: Transl Psychiatry. 2021 Aug 28;11:443. doi: 10.1038/s41398-021-01569-3 (PMC8401367; doi:10.1038/s41398-021-01569-3)
Supplement: Supplementary file 1 — Supplemental Material [file 41398_2021_1569_MOESM1_ESM.docx]

**Supplementary Material for**

**Resilience and personality as predictors of the biological stress load during the first wave of the Covid-19 pandemic in Germany**

Veronika Engert^1,2*^, Jost U. Blasberg^1^, Sophie Köhne^1^, Bernhardt Strauss^1^, Jenny Rosendahl^1^

^1^Institute of Psychosocial Medicine, Psychotherapy and Psychooncology, Jena University Hospital, Friedrich-Schiller University, Jena, Germany

^2^Research Group Social Stress and Family Health, Max Planck Institute for Human Cognitive and Brain Sciences, Leipzig, Germany

^*^Corresponding author: Veronika Engert (veronika.engert@med.uni-jena.de)

**Table of Contents**

[Supplementary Methods 2](#_Toc66887050)

[Supplementary Results 3](#_Toc66887051)

[Table S1. Means (M), standard deviations (SD), and correlations of stress markers (N = 80). 4](#_Toc66887053)

[Table S2. Model indices of multiple regressions for hair cortisol, hair cortisone, and perceived stress with state resilience at T0, state resilience change between T0 and T1, and personality traits as explanatory covariates. 5](#_Toc66887054)

[Table S3. Model indices of multiple regressions for hair cortisol, hair cortisone, and perceived stress with trait resilience at T0, state resilience change between T0 and T1, and personality traits as explanatory covariates. 6](#_Toc66887055)

[Table S4. Model indices of multiple regressions for hair cortisol, hair cortisone, and perceived stress excluding men with state resilience at T0, state resilience change between T0 and T1, and personality traits as explanatory covariates. 7](#_Toc66887056)

[Table S5. Model indices of multiple regressions for hair cortisol, hair cortisone, and perceived stress excluding men with trait resilience at T0 and personality traits as explanatory covariates. 8](#_Toc66887057)

[Supplementary References](#_Toc66887058) 9

# **Supplementary Methods**

**Measures**

Personality characteristics

In the German version^1^ of the NEO Five Factors Inventory (NEO-FFI-30),^2^ participants responded to 30 items (6 items per personality trait) on a five-point Likert scale ranging from 0 “strongly disagree” to 4 “strongly agree”. Mean scores are calculated for each subscale. Higher scores indicate higher levels of extraversion, neuroticism, agreeableness, conscientiousness, and openness to experience.

Resilience

The German version^3^ of the Resilience Scale (RS)^4^ consists of 25 items reflecting two underlying factors (“personal competence” and “acceptance of self and life”; with 17 and eight items, respectively). The items are rated on a seven-point scale ranging from 1 “strongly disagree” to 7 “strongly agree”. All items are worded positively and summed up to a total score with higher scores reflecting higher resilience. The Brief Resilience Scale (BRS)^5^ in its German version^6^ consists of six items rated on a five-point scale ranging from 1 “strongly disagree” to 5 “strongly agree”. After inversion of three negatively phrased items, an average score is calculated. Again, higher scores reflect higher resilience. For both resilience questionnaires, sufficient reliability and validity has been demonstrated.^7^

Subjective stress

In the German version^8^ of the Perceived Stress Scale (PSS-10),^9^ participants rated their stress load on 10 items using a 5-point Likert-scale ranging from 0 “never” to 4 “very often”, and a total sum score was calculated. Higher sum scores indicate a higher level of perceived stress. The PSS-10, also in its German version, has been proven valid and reliable instrument.^8,10^

Hair cortisol and cortisone concentrations

For the assessment of hair cortisol and cortisone concentrations, participants were instructed to take hair strands as close as possible to the scalp from a posterior vertex position. Hair samples were wrapped in aluminum foil and sent to the Institute of Psychosocial Medicine, Psychotherapy and Psychooncology at the Jena University Hospital. There, they were stored in the dark at room temperature until assay at the Department of Psychology, Technische Universität Dresden, Germany. Based on the assumption of an average hair growth rate of 1 cm/month,^11^ we analyzed the proximal 3 cm hair segment to assess cortisol/cortisone accumulation over a 3-month period. Hormone concentrations were measured using liquid chromatography-tandem mass spectrometry (LC–MS/MS), the current gold-standard approach for hair steroid analysis,^12^ following a protocol with a limit of quantification for cortisol and cortisone below 0.09 pg/mg and intra- and inter-assay CVs between 3.7 and 8.8%.^13^

# **Supplementary Results**

## Trait Resilience Multiple Regression Models

Age had a significant positive effect on hair cortisol (*β* = 0.27, 95% CI 0.05 to 0.49, *p* **=** **.019). For hair cortisone, significant positive effects of age** (*β* = 0.25, 95% CI 0.03 to 0.47, *p* **= .028) and extraversion were found** (*β* = 0.27, 95% CI 0.01 to 0.54, *p* **=** **.042), and for perceived stress, a significant positive effect of neuroticism** (*β* = 0.54, 95% CI 0.07 to 0.69, *p* **= .042).**

## **Table S1**. Means (M), standard deviations (SD), and correlations of stress markers (N = 80).

| Variable | *M* | *SD* | 1 | 2 |
| --- | --- | --- | --- | --- |
| 1. log(Cortisol) | 1.61 | 1.20 |  |  |
| 2. log(Cortisone) | 2.53 | 1.08 | .89**  [.84, .93] |  |
| 3. PSS | 16.78 | 6.70 | -.09  [-.31, .13] | -.07  [-.29, .16] |

*Note.* Values in square brackets indicate the 95% confidence interval for each correlation. The confidence interval is a plausible range of population correlations that could have caused the sample correlation (Cumming, 2014). ** *p* < .01.

## **Table S2**. Model indices of multiple regressions for hair cortisol, hair cortisone, and perceived stress with state resilience at T0, state resilience change between T0 and T1, and personality traits as explanatory covariates.

| *Predictors* | *Estimates* | *Std. Beta* | *95% CI* | *Standardized CI* | *P* |
| --- | --- | --- | --- | --- | --- |
|  | **Log(Cortisol)** | | | | |
| Intercept | 1.59 | 0.00 | 1.33 – 1.85 | -0.21 – 0.21 | **<0.001** |
| Age | 0.34 | 0.28 | 0.08 – 0.60 | 0.07 – 0.50 | **0.011** |
| Resilience (BRS T0) | 0.24 | 0.17 | -0.29 – 0.77 | -0.21 – 0.55 | 0.368 |
| Resilience (BRS T1-T0) | -0.16 | -0.14 | -0.55 – 0.22 | -0.48 – 0.19 | 0.402 |
| Neuroticism | 0.44 | 0.37 | 0.09 – 0.79 | 0.08 – 0.66 | **0.014** |
| Extraversion | 0.21 | 0.18 | -0.08 – 0.51 | -0.07 – 0.42 | 0.151 |
| R^2^ / R^2^ adjusted | 0.154 / 0.097 | | | | |
|  | **Log(Cortisone)** | | | | |
| Intercept | 2.53 | 0.00 | 2.30 – 2.76 | -0.21 – 0.21 | **<0.001** |
| Age | 0.28 | 0.26 | 0.05 – 0.52 | 0.04 – 0.49 | **0.020** |
| Resilience (BRS T0) | 0.17 | 0.13 | -0.32 – 0.65 | -0.25 – 0.52 | 0.493 |
| Resilience (BRS T1-T0) | -0.05 | -0.04 | -0.43 – 0.34 | -0.38 – 0.30 | 0.814 |
| Neuroticism | 0.33 | 0.30 | 0.01 – 0.65 | 0.01 – 0.60 | **0.045** |
| Extraversion | 0.27 | 0.25 | 0.00 – 0.53 | 0.00 – 0.50 | **0.049** |
| Agreeableness | -0.18 | -0.17 | -0.42 – 0.06 | -0.39 – 0.05 | 0.131 |
| R^2^ / R^2^ adjusted | 0.155 / 0.085 | | | | |
|  | **Perceived stress (PSS)** | | | | |
| Intercept | 16.83 | 0.00 | 15.68 – 17.98 | -0.17 – 0.17 | **<0.001** |
| Age | 0.86 | 0.13 | -0.33 – 2.04 | -0.05 – 0.31 | 0.156 |
| Resilience (BRS T0) | -0.48 | -0.06 | -2.92 – 1.96 | -0.38 – 0.25 | 0.697 |
| Resilience (BRS T1-T0) | -1.23 | -0.18 | -3.14 – 0.68 | -0.45 – 0.10 | 0.204 |
| Neuroticism | 4.03 | 0.61 | 2.40 – 5.65 | 0.36 – 0.85 | **<0.001** |
| Extraversion | -0.80 | -0.12 | -2.13 – 0.53 | -0.32 – 0.08 | 0.233 |
| Agreeableness | 0.75 | 0.11 | -0.44 – 1.94 | -0.07 – 0.29 | 0.212 |
| Conscientiousness | -0.69 | -0.10 | -1.92 – 0.55 | -0.29 – 0.08 | 0.271 |
| R^2^ / R^2^ adjusted | 0.455 / 0.402 | | | | |

*Note. N* = 80 for all models. Bold *p*-values indicate a significant regression weight at an alpha-level of < .05.

## **Table S3**. Model indices of multiple regressions for hair cortisol, hair cortisone, and perceived stress with trait resilience at T0 and personality traits as explanatory covariates.

| *Predictors* | *Estimates* | *Std. Beta* | *CI* | *Standardized CI* | *P* |
| --- | --- | --- | --- | --- | --- |
|  | **Log(Cortisol)** | | | | |
| Intercept | 1.64 | 0.00 | 1.38 – 1.90 | -0.22 – 0.22 | **<0.001** |
| Age | 0.32 | 0.27 | 0.06 – 0.59 | 0.05 – 0.49 | **0.019** |
| Trait resilience (RS) | 0.08 | 0.07 | -0.31 – 0.48 | -0.26 – 0.40 | 0.675 |
| Neuroticism | 0.28 | 0.23 | -0.09 – 0.64 | -0.07 – 0.54 | 0.135 |
| Extraversion | 0.23 | 0.19 | -0.09 – 0.55 | -0.07 – 0.46 | 0.153 |
| Agreeableness | -0.17 | -0.14 | -0.43 – 0.10 | -0.36 – 0.08 | 0.217 |
| R^2^ / R^2^ adjusted | 0.123 / 0.063 | | | | |
|  | **Log(Cortisone)** | | | | |
| Intercept | 2.55 | 0.00 | 2.32 – 2.78 | -0.21 – 0.21 | **<0.001** |
| Age | 0.27 | 0.25 | 0.03 – 0.51 | 0.03 – 0.47 | **0.028** |
| Trait resilience (RS) | -0.01 | -0.01 | -0.37 – 0.34 | -0.34 – 0.31 | 0.935 |
| Neuroticism | 0.21 | 0.20 | -0.11 – 0.54 | -0.10 – 0.50 | 0.197 |
| Extraversion | 0.29 | 0.27 | 0.01 – 0.58 | 0.01 – 0.54 | **0.042** |
| Agreeableness | -0.21 | -0.19 | -0.44 – 0.03 | -0.41 – 0.03 | 0.087 |
| R^2^ / R^2^ adjusted | 0.139 / 0.081 | | | | |
|  | **Perceived stress (PSS)** | | | | |
| Intercept | 16.84 | 0.00 | 15.69 – 17.99 | -0.17 – 0.17 | **<0.001** |
| Age | 0.82 | 0.12 | -0.37 – 2.01 | -0.06 – 0.30 | 0.174 |
| Trait resilience (RS) | -0.60 | -0.09 | -2.35 – 1.16 | -0.36 – 0.18 | 0.502 |
| Neuroticism | 3.57 | 0.54 | 1.94 – 5.20 | 0.29 – 0.78 | **<0.001** |
| Extraversion | -0.68 | -0.10 | -2.11 – 0.74 | -0.32 – 0.11 | 0.341 |
| Agreeableness | 0.68 | 0.10 | -0.51 – 1.86 | -0.08 – 0.28 | 0.260 |
| R^2^ / R^2^ adjusted | 0.432 / 0.394 | | | | |

*Note. N* = 80 for all models. Bold *p*-values indicate a significant regression weight at an alpha-level of < .05.

## **Table S4**. Model indices of multiple regressions for hair cortisol, hair cortisone, and perceived stress excluding men with state resilience at T0, state resilience change between T0 and T1, and personality traits as explanatory covariates.

|  | **Log(Cortisol)** | | | | |
| --- | --- | --- | --- | --- | --- |
| *Predictors* | *Estimates* | *Std. Beta* | *95% CI* | *Standardized CI* | *p* |
| Intercept | 1.58 | -0.00 | 1.30 – 1.86 | -0.22 – 0.22 | **<0.001** |
| Age | 0.37 | 0.31 | 0.10 – 0.65 | 0.08 – 0.54 | **0.009** |
| Resilience (BRS T0) | 0.21 | 0.15 | -0.41 – 0.82 | -0.29 – 0.58 | 0.505 |
| Resilience (BRS T1-T0) | -0.22 | -0.18 | -0.70 – 0.25 | -0.57 – 0.21 | 0.353 |
| Neuroticism | 0.46 | 0.38 | 0.09 – 0.83 | 0.07 – 0.69 | **0.016** |
| Extraversion | 0.17 | 0.13 | -0.16 – 0.50 | -0.13 – 0.40 | 0.311 |
| R^2^ / R^2^ adjusted | 0.166 / 0.103 | | | | |
|  | **Log(Cortisone)** | | | | |
| Intercept | 2.55 | 0.00 | 2.29 – 2.81 | -0.22 – 0.22 | **<0.001** |
| Age | 0.31 | 0.29 | 0.06 – 0.56 | 0.06 – 0.52 | **0.016** |
| Resilience (BRS T0) | 0.10 | 0.08 | -0.46 – 0.67 | -0.36 – 0.53 | 0.716 |
| Resilience (BRS T1-T0) | -0.17 | -0.14 | -0.65 – 0.30 | -0.53 – 0.25 | 0.475 |
| Neuroticism | 0.34 | 0.31 | -0.01 – 0.68 | -0.01 – 0.63 | 0.054 |
| Extraversion | 0.21 | 0.19 | -0.09 – 0.51 | -0.08 – 0.45 | 0.166 |
| Agreeableness | -0.23 | -0.17 | -0.54 – 0.08 | -0.41 – 0.06 | 0.139 |
| R^2^ / R^2^ adjusted | 0.166 / 0.089 | | | | |
|  | **Perceived stress (PSS)** | | | | |
| Intercept | 16.78 | 0.00 | 15.51 – 18.04 | -0.18 – 0.18 | **<0.001** |
| Age | 1.09 | 0.16 | -0.15 – 2.33 | -0.02 – 0.35 | 0.084 |
| Resilience (BRS T0) | -0.01 | -0.00 | -2.80 – 2.79 | -0.36 – 0.35 | 0.997 |
| Resilience (BRS T1-T0) | -1.30 | -0.18 | -3.64 – 1.03 | -0.49 – 0.14 | 0.268 |
| Neuroticism | 4.57 | 0.69 | 2.83 – 6.32 | 0.42 – 0.95 | **<0.001** |
| Extraversion | -1.20 | -0.17 | -2.67 – 0.28 | -0.38 – 0.04 | 0.109 |
| Agreeableness | 0.24 | 0.03 | -1.28 – 1.77 | -0.16 – 0.22 | 0.752 |
| Conscientiousness | -0.09 | -0.01 | -1.48 – 1.30 | -0.22 – 0.19 | 0.893 |
| R^2^ / R^2^ adjusted | 0.477 / 0.419 | | | | |

*Note. N* = 72 for all models. Bold *p*-values indicate a significant regression weight at an alpha-level of < .05.

## **Table S5**. Model indices of multiple regressions for hair cortisol, hair cortisone, and perceived stress excluding men with trait resilience at T0 and personality traits as explanatory covariates.

|  | **Log(Cortisol)** | | | | |
| --- | --- | --- | --- | --- | --- |
| *Predictors* | *Estimates* | *Std. Beta* | *95% CI* | *Standardized CI* | *p* |
| Intercept | 1.66 | -0.00 | 1.37 – 1.94 | -0.23 – 0.23 | **<0.001** |
| Age | 0.34 | 0.29 | 0.06 – 0.62 | 0.05 – 0.52 | **0.017** |
| Trait resilience (RS) | 0.18 | 0.15 | -0.27 – 0.62 | -0.22 – 0.52 | 0.429 |
| Neuroticism | 0.32 | 0.27 | -0.07 – 0.72 | -0.06 – 0.60 | 0.106 |
| Extraversion | 0.15 | 0.12 | -0.22 – 0.52 | -0.18 – 0.41 | 0.424 |
| Agreeableness | -0.19 | -0.13 | -0.53 – 0.15 | -0.36 – 0.10 | 0.264 |
| R^2^ / R^2^ adjusted | 0.136 / 0.071 | | | | |
|  | **Log(Cortisone)** | | | | |
| Intercept | 2.57 | 0.00 | 2.31 – 2.82 | -0.23 – 0.23 | **<0.001** |
| Age | 0.28 | 0.26 | 0.03 – 0.53 | 0.03 – 0.50 | **0.028** |
| Trait resilience (RS) | 0.03 | 0.03 | -0.37 – 0.43 | -0.35 – 0.40 | 0.887 |
| Neuroticism | 0.22 | 0.20 | -0.14 – 0.57 | -0.13 – 0.53 | 0.224 |
| Extraversion | 0.23 | 0.20 | -0.10 – 0.56 | -0.09 – 0.50 | 0.174 |
| Agreeableness | -0.24 | -0.18 | -0.55 – 0.06 | -0.42 – 0.05 | 0.117 |
| R^2^ / R^2^ adjusted | 0.139 / 0.074 | | | | |
|  | **Perceived stress (PSS)** | | | | |
| Intercept | 16.82 | 0.00 | 15.57 – 18.06 | -0.18 – 0.18 | **<0.001** |
| Age | 0.96 | 0.14 | -0.27 – 2.18 | -0.04 – 0.33 | 0.124 |
| Trait resilience (RS) | 0.04 | 0.01 | -1.92 – 2.00 | -0.29 – 0.30 | 0.965 |
| Neuroticism | 4.01 | 0.60 | 2.27 – 5.74 | 0.34 – 0.86 | **<0.001** |
| Extraversion | -1.13 | -0.16 | -2.76 – 0.50 | -0.40 – 0.07 | 0.172 |
| Agreeableness | 0.21 | 0.03 | -1.29 – 1.71 | -0.16 – 0.21 | 0.777 |
| R^2^ / R^2^ adjusted | 0.456 / 0.415 | | | | |

*Note. N* = 72 for all models. Bold *p*-values indicate a significant regression weight at an alpha-level of < .05.

# **Supplementary References**

1. Körner A, et al. Efficient and valid assessment of personality traits: Population norms of a brief version of the NEO Five-Factor Inventory (NEO-FFI). *Arch Psychiatry Psychother*. 2015;17(1):21–32. doi: 10.12740/APP/36086
2. Costa PT, McCrae RR. *Revised NEO personality inventory (NEO-PI-R) and NEO five-factor inventory (NEO-FFI). Professional manual*. Odessa, FL: Psychological Assessment Ressources; 1992.
3. Schumacher J, Leppert K, Gunzelmann T, Strauß B, Brähler E. Die Resilienzskala – Ein Fragebogen zur Erfassung der psychischen Widerstandsfähigkeit als Personmerkmal [The Resilience Scale - A questionnaire to assess resilience as a personality characteristic]. *Z Klin Psychol Psychiatr Psychother*. 2005;53:16–39.
4. Wagnild GM, Young HM. Development and psychometric evaluation of the Resilience Scale. *J Nurs Meas*. 1993;1(2):165-78.
5. Smith BW, et al. The brief resilience scale: assessing the ability to bounce back. *Int J Behav Med*. 2008;15(3):194-200. doi: 10.1080/10705500802222972.
6. Chmitorz A, et al. Population-based validation of a German version of the Brief Resilience Scale. *PLoS One*. 2018;13(2):e0192761. doi: 10.1371/journal.pone.0192761.
7. Windle G, Bennett KM, Noyes J. A methodological review of resilience measurement scales. *Health Qual Life Outcomes*. 2011;9:8. doi: 10.1186/1477-7525-9-8.
8. Klein EM, et al. The German version of the Perceived Stress Scale - psychometric characteristics in a representative German community sample. *BMC Psychiatry*. 2016;16:159. Published 2016 May 23. doi:10.1186/s12888-016-0875-9
9. Cohen S, Kamarck T, Mermelstein R. A global measure of perceived stress. *J Health Soc Behav*. 1983;24:385–96. doi: 10.2307/2136404.
10. Cohen S, Williamson G. Perceived stress in a probability sample of the United States. In Spacapan S, Oskamp S, eds. *The social psychology of health: Claremont symposium on applied social psychology*. Newbury Park, CA: Sage; 1988:31-67.
11. Wennig R. Potential problems with the interpretation of hair analysis results. *Forensic Sci Int*. 2000;107(1-3):5-12. doi:10.1016/s0379-0738(99)00146-2
12. Gao W, Kirschbaum C, Grass J, Stalder T. LC-MS based analysis of endogenous steroid hormones in human hair. *J Steroid Biochem Mol Biol*. 2016;162:92-99. doi:10.1016/j.jsbmb.2015.12.022
13. Gao W, et al. Quantitative analysis of steroid hormones in human hair using a column-switching LC-APCI-MS/MS assay. *J Chromatogr B Analyt Technol Biomed Life Sci*. 2013;928:1-8. doi:10.1016/j.jchromb.2013.03.008
